# Angiotensin II-independent abnormal renal vascular reactivity during puromycin nephropathy

**DOI:** 10.25122/jml-2023-0367

**Published:** 2024-03

**Authors:** Luis Isaias Juncos, Akinwunmi Oluwaseun Adeoye, Fernando Luis Martin, Julio Pedro Juncos, Sandra Teresita Baigorria, Néstor Horacio García

**Affiliations:** 1 Department of Renal Physiology, J. Robert Cade Foundation, Córdoba, Argentina; 2 Department of Biochemistry, Federal University Oye-Ekiti, Ekiti state, Nigeria; 3 Department of Renal Physiology, INICSA-CONICET, Córdoba, Argentina

**Keywords:** kidney, puromycin nephropathy, quinapril, sodium, glomerulonephritis

## Abstract

Experimental glomerulonephritis results in hypertension that is sensitive to salt. Nevertheless, salt retention alone cannot explain the increase in blood pressure. Angiotensin antagonistic therapy reduces hypertension caused by puromycin amino nucleosides (PAN). We investigated the hypothesis that PAN modifies renal vascular reactivity through processes dependent on angiotensin. Long-Evans rats were given an intraperitoneal injection of either puromycin (150 mg/kg) or saline (controls). Group 1 was fed a normal sodium diet (NSD, *n* = 9). Group 2 was given 30 mg/L of quinapril (Q) in addition to NSD (NSD + Q; *n* = 6). Group 3 received a high sodium diet (HSD, *n* = 7), and Group 4 received HSD + Q (*n* = 7). Systolic blood pressure (SBP), plasma creatinine, proteinuria, and sodium balance were monitored for 12 days. On day 15, renal vascular reactivity was assessed by administering increasing doses of angiotensin II, acetylcholine (ACh), and sodium nitroprusside (SNP) directly into the renal artery. SBP progressively increased in all PAN groups. This increase in SBP was greater in the HSD groups and was not significantly altered by Q treatment. SBP increased by 22 ± 4% (NSD), 51 ± 5% (NSD + Q), 81 ± 10% (HSD), and 65 ± 8% (HSD + Q). The renal blood flow of PAN rats did not return to baseline despite their normal renal vasoconstrictor responses to angiotensin II. Additionally, they showed reduced renal vasodilator responses to SNP and Ach. The vasodilator responses to both vasodilators were surprisingly unaffected by the inhibition of the angiotensin-converting enzyme (ACE). Renal vasodilator responses to both endothelium-dependent and independent variables were reduced in early PAN-induced hypertension. We found that the angiotensin-mediated mechanism is not responsible for this altered renal vasoreactivity.

## INTRODUCTION

Arterial hypertension is a frequent clinical feature of glomerular disease [1-4]. Its importance stems from the fact that it is not only a result of the primary disease but also a major risk factor that determines the likelihood and progression rate of renal disease [5-9]. Because of this, it is now recommended that we aggressively strive to lower blood pressure levels to less than 120/75 mmHg in patients with chronic glomerular disease and proteinuria [10]. However, achieving this degree of blood pressure control can be challenging at times. This may be due to the presence of various concurrent mechanisms that trigger systemic hypertension during glomerulonephritis. Consequently, it is important to understand these mechanisms and their role in the development of hypertension. One important mechanism proposed in the development of hypertension is volume expansion secondary to excessive sodium retention [11-15]. Glomerulonephritis often presents as a salt-sensitive model of hypertension, and enhanced renal sodium reabsorption has been reported [16]. However, the presence of sodium retention in the absence of hypertension in numerous situations suggests that additional mechanisms are necessary for hypertension to manifest. One potential mechanism is via the renin-angiotensin system (RAS). High plasma renin activity (PRA) has been reported in some studies [17,18], and short-term treatment with an angiotensin-converting enzyme (ACE) inhibitor reportedly lowers blood pressure in patients with acute nephritis [19]. Thus, we hypothesized that early glomerulonephritis is associated with altered renal vascular reactivity via an angiotensin II-dependent mechanism. In addition, we evaluated whether salt intake interacts with angiotensin II and aggravates abnormal vascular reactivity. For this, we studied renal vascular reactivity in rats with early experimental glomerulonephritis induced by puromycin amino nucleoside (PAN) fed a normal or high salt diet and treated with an ACE inhibitor or vehicle.

## MATERIAL AND METHODS

Young male Long-Evans rats (120 to 150 g) received normal sodium (0.8%) Purina rat chow for 7 days. We then placed the rats into metabolic cages to collect 24-hour urine specimens for measuring baseline sodium, creatinine, and protein excretion rates. Immediately after completing the urine collections, we drew blood samples to measure serum sodium and plasma creatinine, and we determined systolic blood pressure (SBP) using the tail-cuff method. Rats were then randomized to receive either a single intraperitoneal (I.P.) injection of 100 µL normal saline (Sham) or puromycin (15 mg/100 g body weight; Sigma) to induce PAN. Injecting this dose of puromycin induces heavy proteinuria within 7 days. Rats with PAN nephropathy were then divided into four groups. **Group 1** received a normal sodium diet (0.8%), **group 2** a normal sodium diet plus quinapril (30 mg/L in their drinking water), **group 3** received a high sodium diet (4%), and **group 4** a high sodium diet plus quinapril. This dose of quinapril has previously been shown to inhibit ACE effectively. All baseline studies were repeated on the 14^th^ day after the injection. On day 15, rats were anesthetized with sodium pentobarbital (40 mg/kg). The femoral artery was cannulated to monitor mean arterial pressure (MAP) continuously. An electromagnetic flow probe was placed around the left renal artery through a mid-abdominal incision to measure renal blood flow (RBF) continuously. A perfusion needle was positioned via the abdominal aorta into the left renal artery. Following a 30-minute stabilization period for MAP and RBF, bolus injections of increasing doses (10^-8^ to 10^-5^ mol/L) of angiotensin II, acetylcholine (Ach), and sodium nitroprusside (SNP) were administered through a perfusion needle while monitoring MAP and RBF. The dose of each drug was injected three times with 20-minute intervals between injections. Changes in MAP and RBF were averaged and presented as percentages in dose-response curves.

### Statistics

All results are expressed as mean ± standard error. Paired t-tests were used to compare differences between means at baseline and the end of the study. Comparisons between multiple groups, both at baseline and at the end of the study, were performed using one-way analysis of variance (ANOVA) followed by Dunnett's multiple comparison post-hoc test. One-way repeated measures ANOVA was used to compare the RBF changes induced by angiotensin II, Ach, and SNP. Values of *P* <0.05 were considered significant.

## RESULTS

### Blood pressure and renal function

**Figure 1:**
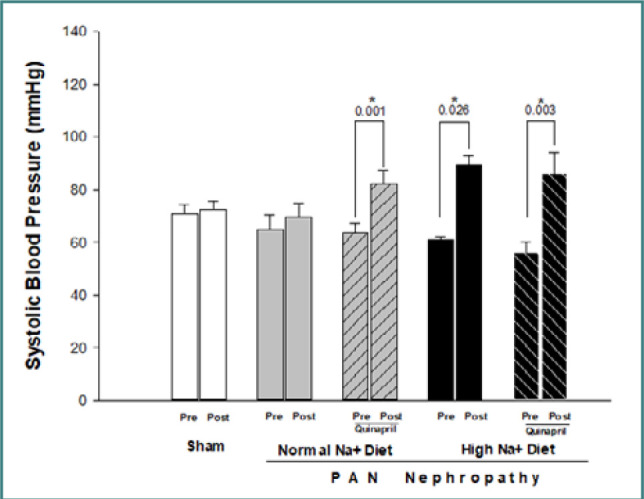
Effects of quinapril on SBP in rats with PAN nephropathy on normal (0.8%) and high (4%) sodium diets. Measurements were taken before (Pre) and 14 days after (Post) administering a single intraperitoneal injection of PAN (15 mg/100 g body weight).

Changes in SBP in all groups are depicted in Figure 1. The SBP of rats with PAN nephropathy on a normal sodium diet (Group 1) was 65.0 ± 5.4 mmHg at the beginning and 71.7 ± 4.45 mmHg at the end of the study. A high sodium diet significantly increased blood pressure (SBP increased from 60.8 ± 1.2 to 101.4 ± 10.2 mmHg; *P* <0.05). Quinapril did not prevent the increase in blood pressure in rats with PAN nephropathy. In rats on a high sodium diet, quinapril treatment led to an increase in SBP from 56.2 ± 4.0 to 92.1 ± 6.7 mmHg (*P* <0.003). Interestingly, rats on a normal sodium diet had a greater increase in SBP with quinapril (from 63.5 ± 3.7 to 88.2 ± 5.4 mmHg; *P* <0.001).

**Figure 2:**
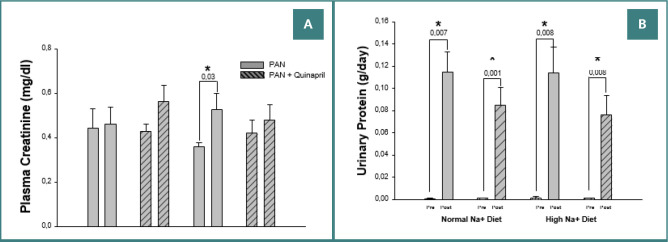
Comparative effects of quinapril on (A) plasma creatinine and (B) urinary protein excretion in rats with pan nephropathy on normal (0.8%) and high (4%) sodium diets. Measurements were taken before (Pre) and 14 days after (Post) administering a single intraperitoneal injection of PAN (15 mg/100 g body weight).

Plasma creatinine did not change significantly in any group in the short span of these experiments (Figure 2A). In contrast, the protein excretion rate increased in all the PAN nephropathy groups (Figure 2B). The sodium excretion rate also increased significantly despite net sodium retention in all experimental groups, as is the typical course in PAN nephropathy. Quinapril treatment made no difference in the rate of increase in sodium excretion. The cumulative sodium balances at the end of the study were 0.78, 0.29, 47.6, and 36.4 mEq for groups 1, 2, 3, and 4, respectively. These results were consistent with the physical aspects of rats with PAN nephropathy; that is, the ones on the high salt diet had marked ascites, whereas the ones on the normal sodium diet did not.

### Renal vascular reactivity

**Figure 3:**
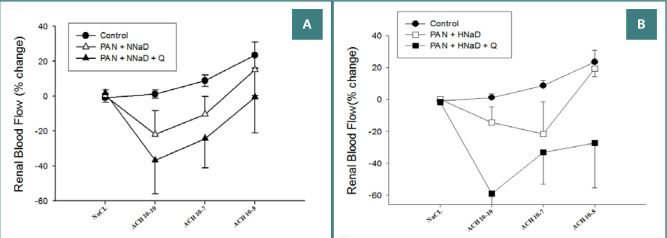
Effects of quinapril on renal blood flow response to intrarenal administration of increasing doses of the endothelium-dependent vasodilator Ach in rats with PAN nephropathy. A, results in rats on a normal sodium diet (0.8%); B, results on a high sodium diet (4%).

**Figure 4:**
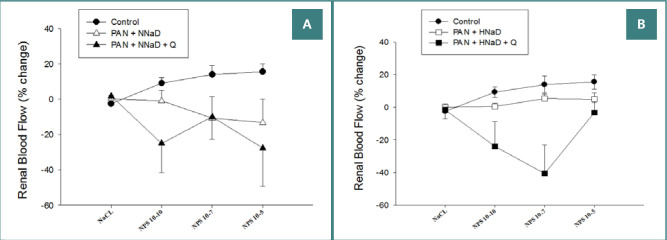
Effects of quinapril on renal blood flow response to intrarenal administration of increasing doses of the endothelium-independent vasodilator NPS in rats with PAN nephropathy. A, results in rats on the normal sodium diet (0.8%); B, results on a high sodium diet (4%).

As presented in Figures 3 and 4, renal vascular responses to endothelium-dependent or independent vasodilators were impaired. Figure 3 shows Ach-induced responses in PAN nephropathy and sham rats maintained on either a normal sodium diet (Figure 3A) or a high sodium diet (Figure 3B). Ach caused dose-dependent vasodilation in the sham group, which did not decrease during the study. In contrast, Ach had no effect (PAN nephropathy rats on a high sodium diet) or caused variable degrees of vasoconstriction (PAN nephropathy rats on a normal sodium diet). Quinapril did not preserve endothelial-dependent vasodilation in rats with PAN nephropathy on a normal or high sodium diet. Ach continued to have no effect or induce variable degrees of vasoconstriction. Similar to the endothelium-dependent responses, PAN nephropathy also altered the response to the endothelium-independent vasodilator, SNP (Figure 4). In the normal sodium groups (Figure 4A), SNP tended to cause paradoxical vasoconstriction that was not altered by quinapril. Similarly, in the high sodium group (Figure 4B), SNP did not cause renal vasodilation. Although responses to SNP in the quinapril-treated group were variable, quinapril did not improve renal vasodilatory responses to this agent.

**Figure 5:**
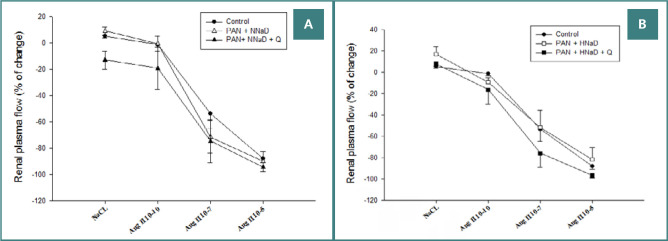
Effects of quinapril on renal blood flow response to intrarenal administration of increasing doses of the endothelium-independent vasodilator ANG in rats with PAN nephropathy. A, the results in rats on a normal sodium diet (0.8%); B, results on a high sodium diet (4%).

Our findings highlight that RBF did not completely return to baseline in rats with PAN nephropathy after discontinuing angiotensin II, perhaps suggesting altered vasodilation. The intrarenal administration of increasing doses of angiotensin II caused progressive renal vasoconstriction that was similar in all groups, including the sham group (Figure 5A,B). These findings suggest that the renal vascular response to angiotensin was unaltered early during PAN nephropathy. Early PAN nephropathy-induced hypertension was associated with blunted renal vasodilator responses to both endothelium-dependent and independent factors. Hypertension, but not the altered vascular reactivity, was exacerbated by high salt intake. Both alterations were not dependent on angiotensin-mediated mechanisms.

## DISCUSSION

This study demonstrated that early in the PAN nephropathy model, glomerulonephritis induced both salt-sensitive hypertension and altered vascular reactivity. This alteration in vascular reactivity appears to be different from that usually seen in experimental hypertension, as the primary defect was manifested as altered endothelial-independent vasodilation. In addition, the altered vascular reactivity was not affected by sodium intake, although high sodium intake worsened hypertension. Finally, inhibition of ACE did not exert a beneficial effect on either hypertension or altered vascular reactivity, suggesting that RAS was not playing a major role in the pathogenesis of these abnormalities.

Administration of a single dose of PAN induces salt-sensitive hypertension associated with nephropathy, which can be divided into several phases. Early PAN nephropathy is characterized by heavy proteinuria with variable degrees of impaired glomerular filtration. Proteinuria gradually resolves, but there is a persistence of impaired function and glomerular hypertension. Finally, in the late phase, there is extensive glomerular sclerosis with recurrent proteinuria. In the present study, we focused on the abnormalities in the early phase of PAN nephropathy. These rats developed early-onset salt-sensitive hypertension. While the absolute blood pressure levels were not very high, it is important to note that these rats were very young and that the blood pressures were consistent with the age-matched sham rats. Induction of PAN nephropathy in rats maintained on a normal sodium diet did not significantly alter blood pressure. However, it caused a striking increase in blood pressure (81 ± 10%) in the rats fed a high salt diet associated with a marked positive sodium balance and ascites.

In our study, salt-sensitive hypertension observed in rats with PAN nephropathy was expected and aligns well with findings from previous studies, demonstrating the reliability of our PAN nephropathy model. While sodium retention typically does not always lead to increased blood pressure often due to compensatory peripheral vasodilation, we hypothesized that an alternative mechanism might facilitate the blood pressure response to elevated salt intake. Our investigations focused on abnormal renal vascular reactivity as a potential underlying factor.

Indeed, altered vascular reactivity tends to be present in many forms of experimental and human hypertension. However, it is usually characterized by abnormal endothelium-dependent vasodilation and/or enhanced vasoconstriction. We found that PAN nephropathy did not affect vasoconstriction, but the vessels did not vasodilate to their basal level after receiving Ang II. Given that PAN also impaired the renal vascular responses to endothelial-dependent and independent vasodilators, it suggests that PAN might have a direct toxic effect on the renal vascular smooth muscle that predominantly impairs vasodilation. It is interesting to note that this pattern of impaired vascular reactivity was different from that usually observed during hypertension and was present in all of the groups, including in rats with PAN nephropathy fed a normal sodium diet, which was not hypertensive. Thus, our data suggest that PAN nephropathy was associated with a renal vascular defect that seems to be distinct from that seen during hypertension and that this defect could give rise to salt sensitivity.

There are several potential mechanisms by which salt-sensitive hypertension can be precipitated. Much attention has been paid to activating the RAS system. This is because of its prominent role in the regulation of renal hemodynamics and salt and water excretion. Indeed, infusion of a low dose of Ang II leads to salt-sensitive hypertension, and Ang II-mediated sodium retention has been suspected in various forms of glomerulonephritis. For instance, plasma renin activity has been elevated in human glomerulonephritis and various experimental models of glomerular disease [17,18,20,21]. Moreover, angiotensinogen concentration was elevated before the appearance of proteinuria in the PAN nephropathy model. Further evidence for the role of the RAS was advanced by Hisada *et al*. [22] in an anti-glomerular basement membrane (anti-GBM) glomerulonephritis model induced in Ang II type 1a receptor-deficient mice. They showed that only angiotensin II type 1 receptor (AT1a ^+/+^) mice develop proteinuria and hypertension, even though AT1a^-/-^ mice had similar aggregation of autologous antibodies [23]. Finally, Parra *et al*. [19] showed a rapid control of hypertension by the ACE inhibitor Captopril in patients with acute glomerulonephritis. However, our findings do not support the role of an Ang II-dependent mechanism because quinapril did not improve any of the variables measured. Blood pressure elevation and the positive cumulative sodium balance were unchanged. Likewise, mean serum creatinine and mean urinary protein excretion were unchanged. Finally, quinapril did not improve the abnormal renal vascular reactivity induced by PAN in either of the diets. In agreement with these findings, Anderson *et al*. [24] have previously shown that proteinuria and renal function were not improved with enalapril during the early phase of PAN nephropathy. Ichikawa *et al*. [25] showed that saralasin increases glomerular filtration rate (GFR) and single-nephron GFR during unilateral puromycin nephritis but does not improve unilateral sodium excretion. Similarly, Pedraza-Chaverri *et al*. [26] showed no correlation between sodium retention and plasma renin activity or concentration in patients with nephritis. The most remarkable findings, however, were reported by Gabbai *et al*. [27]. These investigators found that quinapril corrected hypertension associated with 2-kidney, 1-clip Goldblatt hypertensive rats but did not lower the blood pressure if the same model was superimposed with anti-GBM-induced glomerulonephritis [27]. These studies suggest that RAS may play a role in hypertension present in certain forms of glomerulonephritis or the chronic phases but does not appear to play a role in either hypertension, renal dysfunction, or abnormal renal vascular responses present during early PAN nephropathy.

Our investigation has limitations regarding the molecular mechanism involved in the vascular and tubular response to puromycin-induced nephropathy. We aimed to evaluate the physiological response of a kidney damaged by glomerulonephritis to sodium load in an inhibited renin-angiotensin system.

## Conclusion

Our study showed that PAN nephropathy-induced salt-sensitive arterial hypertension was accompanied by decreased renal vasodilator responses to both endothelium-dependent and endothelium-independent stimuli, which was not likely secondary to hypertension. In contrast to other models of hypertension, blocking RAS in PAN nephropathy does not improve hypertension, proteinuria, or renal vascular reactivity. Therapies aimed at reducing the sodium load and improving the altered vascular reactivity may be particularly useful in managing these patients.

## References

[1] Johnston PA, Davison AM (1993). Hypertension in adults with idiopathic glomerulonephritis and normal serum creatinine. A report from the MRC Glomerulonephritis Registry. Nephrol Dial Transplant.

[2] Marcus VN, Corpa VS (2002). Systemic hypertension in patients with glomerulonephritis. Ren Fail.

[3] Floege J, Johnson RJ, Feehally J (2010). Introduction to glomerular disease. Compr Clin Nephrol.

[4] Qian Q, Nasr SH (2014). Diagnosis and treatment of glomerular diseases in elderly patients. Adv Chronic Kidney Dis.

[5] Cameron JS (2015). Hypertension in chronic glomerulonephritis. Electrolyte Blood Press.

[6] Ritz E, Rambausek M, Hasslacher C, Mann J (1989). Pathogenesis of hypertension in glomerular disease. Am J Nephrol.

[7] Tikkanen I, Fyhrquist M, Miettinen A, Tornroth T (1980). Autologous immune complex nephritis and DOCA-NaCl load: a new model of hypertension. Acta Pathol Microbiol Scand Sect A Pathol.

[8] Wenzel UO, Thaiss F, Panzer U, Schneider A, Schwietzer G, Helmchen U (1999). Effect of renovascular hypertension on experimental glomerulonephritis in rats. J Lab Clin Med.

[9] Webster AC, Nagler EV, Morton RL, Masson P (2017). Chronic kidney disease. Lancet.

[10] (1997). The sixth report of the Joint National Committee on the prevention detection, evaluation, and treatment of high blood pressure. Arch Intern Med.

[11] Birkenhäger WH, Schalekamp MA, Schalekamp-Kuyken MP, Kolsters G, Krauss XH (1970). Interrelations between arterial pressure, fluid-volumes, and plasma-renin concentration in the course of acute glomerulonephritis. Lancet.

[12] Rodríguez-Iturbe B, Baggio B, Colina-Chourio J, Favaro S, García R, Sussana F (1981). Studies on the renin-aldosterone system in the acute nephritic syndrome. Kidney Int.

[13] Zolty E, Ibnou-Zekri N, Izui S, Feraille E, Favre H (1999). Glomerulonephritis and sodium retention: enhancement of Na+/K+-ATPase activity in the collecting duct is shared by rats with puromycin induced nephritic syndrome and mice with spontaneous lupus-like glomerulonephritis. Nephrol Dial Transplant.

[14] Gavras I, Gavras H (2012). Volume-expanded hypertension: the effect of fluid overload and the role of the sympathetic nervous system in salt-dependent hypertension. J Hypertens.

[15] Chamarthi B, Williams JS, Williams GH (2010). A mechanism for salt-sensitive hypertension: abnormal dietary sodium mediated vascular response to angiotensin II. J Hypertens.

[16] Chachati A, Gordon JP (1985). Impaired sodium excretion in experimental glomerulonephritis: an explanation for the controversies in the literature. Ren Physiol.

[17] Medina A, Davies D, Brown J, Fraser R (1974). A study of the renin-angiotensin system in the nephritic syndrome. Nephron.

[18] Boer P, Roos JC, Geyskes GG, Dorhout-Mees EJ (1980). Observations on plasma renin substrate in the nephritic syndrome. Nephron.

[19] Parra G, Rodriguez-Iturbe B, Colina-Chourio J, Garcia R (1988). Short-term treatment with Captopril in hypertension due to acute glomerulonephritis. Clin Nephrol.

[20] Chonko AM, Bay WH, Stein JH, Ferris TF (1977). The role of renin and aldosterone in the salt retention of edema. Am J Med.

[21] Imai M, Sokabe H (1968). Plasma renin and angiotensinogen levels in pathological states associated with edema. Arch Dis Child.

[22] Yayama K, Konishi K, Ohta A, Takano M, Ohtani R, Itoh N (1993). Elevation of plasma angiotensinogen in rats with experimentally induced nephrosis. Nephron.

[23] Hisada Y, Sugaya T, Yamanouchi M, Uchida H (1999). Angiotensin II plays a pathogenic role in immune-mediated renal injury in mice. J Clin Invest.

[24] Anderson S, Diamond JR, Karnovsky MJ, Brenner BM (1988). Mechanisms underlying transition from acute glomerular injury to late glomerular sclerosis in a rat model of nephritic syndrome. J Clin Invest.

[25] Ichikawa I, Rennke HG, Hoyer JR (1983). Role for the intrarenal mechanisms in the impaired salt excretion of experimental nephrotic syndrome. J Clin Invest.

[26] Pedraza CJ, Cruz C, Ibarra RM (1990). Pathophysiology of experimental nephritic syndrome induced by puromycin aminonucleoside in rats. The role of proteinuria hypoproteinemia and the renin-angiotensin-aldosterone system on sodium retention. Rev Invest Clin.

[27] Gabbai FB, De Nicola L, Peterson OW (1996). Renal response to blood pressure elevation in normal and glomerulonephritic rats. J Am Soc Nephrol.

